# A 3D Bioprinted in vitro Model of Neuroblastoma Recapitulates Dynamic Tumor‐Endothelial Cell Interactions Contributing to Solid Tumor Aggressive Behavior

**DOI:** 10.1002/advs.202200244

**Published:** 2022-05-29

**Authors:** Liqun Ning, Jenny Shim, Martin L. Tomov, Rui Liu, Riya Mehta, Andrew Mingee, Boeun Hwang, Linqi Jin, Athanasios Mantalaris, Chunhui Xu, Morteza Mahmoudi, Kelly C. Goldsmith, Vahid Serpooshan

**Affiliations:** ^1^ Wallace H. Coulter Department of Biomedical Engineering Emory University School of Medicine and Georgia Institute of Technology Atlanta GA 30332 USA; ^2^ Department of Pediatrics Emory University School of Medicine Atlanta GA 30322 USA; ^3^ Aflac Cancer and Blood Disorders Center Children's Healthcare of Atlanta Atlanta GA 30342 USA; ^4^ Children's Healthcare of Atlanta Atlanta GA 30322 USA; ^5^ Department of Biology Emory University Atlanta GA 30322 USA; ^6^ Department of Radiology and Precision Health Program Michigan State University East Lansing MI 48824 USA

**Keywords:** embedded 3D bioprinting, endothelial cell, neuroblastoma, tumor growth and invasion, tumor microenvironment, vascularized model

## Abstract

Neuroblastoma (NB) is the most common extracranial tumor in children resulting in substantial morbidity and mortality. A deeper understanding of the NB tumor microenvironment (TME) remains an area of active research but there is a lack of reliable and biomimetic experimental models. This study utilizes a 3D bioprinting approach, in combination with NB spheroids, to create an in vitro vascular model of NB for exploring the tumor function within an endothelialized microenvironment. A gelatin methacryloyl (gelMA) bioink is used to create multi‐channel cubic tumor analogues with high printing fidelity and mechanical tunability. Human‐derived NB spheroids and human umbilical vein endothelial cells (HUVECs) are incorporated into the biomanufactured gelMA and cocultured under static versus dynamic conditions, demonstrating high levels of survival and growth. Quantification of NB‐EC integration and tumor cell migration suggested an increased aggressive behavior of NB when cultured in bioprinted endothelialized models, when cocultured with HUVECs, and also as a result of dynamic culture. This model also allowed for the assessment of metabolic, cytokine, and gene expression profiles of NB spheroids under varying TME conditions. These results establish a high throughput research enabling platform to study the TME‐mediated cellular‐molecular mechanisms of tumor growth, aggression, and response to therapy.

## Graphical abstract

Manufacturing a biomimetic, highly tunable, 3D in vitro model using embedded bioprinting strategy (A) in combination with neuroblastoma spheroids and human endothelial cells (B). The established tumor microenvironment model can serve as a high‐throughput research enabling platform to generate insights into the neuroblastoma tumor‐endothelial cell interactions, as well as tumor growth and aggression at the cellular and molecular levels (C‐D).

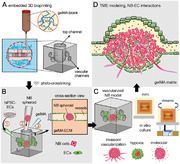

## 1 Introduction

Neuroblastoma (NB) is the most common extracranial solid tumor of childhood arising from the developing sympathetic nervous system and accounts for approximately 15% of all pediatric oncology deaths.^[^ ^1^ ^]^ Despite intense multimodal therapies encompassing chemotherapy, surgery, myeloablative high dose chemotherapy followed by autologous stem cell rescue, radiation, and immunotherapy, more than half of patients with high‐risk NB relapse with incurable disease.^[^ ^2^ ^]^ Over the years, there has been increasing investigation into the importance of the NB tumor microenvironment (TME) and its influence on therapy response and tumor behavior.^[^ ^3^ ^]^ Although, in vivo mouse xenograft models of NB have been the most effective for drug testing and validations, the lack of effective in vitro or *ex vivo* platforms that recapitulate the NB TME has been a barrier to efficient and real‐time dynamic studies.^[^ ^4^ ^]^ The recognized demand for incorporating multiscale complexity to mimic tumor heterogeneity has motivated the development of in vitro models that can recapitulate the physical and biological functions of the native TME.^[^ ^5^ ^]^

A variety of biofabrication strategies have been employed to create in vitro 3D models of human tissues, including casting, freeze‐drying, microfluidic platforms, porous polymeric microparticles, photolithography, electrospinning, and 3D bioprinting.^[^ ^6^ ^]^ Among these techniques, 3D printing and bioprinting have received increasing attention for engineering various TME mimics.^[^ ^7^ ^]^ 3D bioprinting is an additive manufacturing technique that uses hydrogels and cells as bioinks to build 3D structures following the designed models, holding a promising perspective for human disease modeling.^[^ ^8^ ^]^ Unique advantages of bioprinting methods to create cancer models include the robust spatial control on cell‐biomaterial deposition which enables the creation of highly complex and heterogenous 3D structures, the personalized medicine features, and the ability to incorporate a perfusable vascular network to closely mimic the often highly vascularized tumor tissue.^[^ ^7a,b^ ^]^ Bioprinting tumor models, however, face several challenges, including the relatively slow and complex process of manufacturing for some printing methods, and the still limited number of available bioinks that can closely mimic the extracellular matrix (ECM) within the TME.^[^ ^6^, ^9^ ^]^

Both tumor and stromal cells have been successfully bioprinted either separately or collectively in predesigned spaces to mimic the native tumor.^[^ ^10^ ^]^ By precisely adjusting the bioink properties during and post printing, a variety of 3D cancer models have been developed.^[^ ^4^, ^11^ ^]^ Tumor cells incorporated within these printed models demonstrated the processes of extension, migration (invasion), and forming clusters in co‐culture with endothelial cells (ECs) and showed cellular functions and drug responses comparable with those reported in vivo.^[^ ^11b,c,e^ ^]^ Nevertheless, current bioprinted cancer models still face several significant challenges which include 1) low structural fidelity and reproducibility due to the inherent physical and biological properties of hydrogel bioinks; 2) low printed cell density which is far less than the physiological tumor circumstance; and 3) relatively simple tissue architectures which rarely recapitulate the complex, dynamic, and vascularized TME.^[^ ^12^ ^]^ These hurdles have limited the capacity of current bioprinted models for in‐depth analysis of the mechanisms underlying cancer progression and response to therapy,^[^ ^4^, ^13^ ^]^ raising the great need for creating more reproducible and robust vascularized TME platforms.

NB cells cultured as spheroids have been shown to better mimic the growth characteristics of in vivo solid tumors.^[^ ^11^, ^14^ ^]^ However, this spheroid model alone cannot fully exhibit the complexity of the TME which integrates multiple other cellular components including ECs, as well as the tissue ECM, which has been shown to modulate both chemotherapy response and expression of key oncogenes relevant to NB such as *MYCN*.^[^ ^15^ ^]^ Recently, the formation of 3D multicellular tumor spheroids that preserve the cell‐cell and cell‐environment interactions have been progressively used to recapitulate the natural 3D TMEs.^[^ ^16^ ^]^ By mixing ECs with cancer cells, 3D hybrid cancer spheroids exhibit some of the major hallmarks of cancers including cancer invasion and angiogenesis.^[^ ^16^ ^]^ The major drawback of this strategy is its limited physiological relevance, where the hierarchical pattern of a TME is missed by simply mixing endothelial cells and cancer cells. To study tumor invasion, metastasis, and therapeutic responses in a dynamic environment, microfluidic‐based devices or microvessel networks have been developed and have shown great potential and made considerable outcomes.^[^ ^11d^ ^]^ However, these models require multiple post‐manufacturing steps and precise geometrical control, which might be harmful for the living cells and restrict the reproducibility of models for high throughput screening.^[^ ^17^ ^]^

This study aims to develop a vascularized in vitro model of NB to serve as a high‐throughput, reproducible platform for investigations of the cellular and molecular mechanisms of tumor progression, invasion, and response to therapy. A hydrogel‐base cubic model with multiple vascular channels was designed and manufactured using the embedded 3D bioprinting technique. Through systematic adjustment of the printing parameters based on the measured rheological properties of both gelatin methacrylate (gelMA) bioink and the support Carbopol bath, complex 3D constructs with high structural fidelity and reproducibility were fabricated. GelMA crosslinking was intricately regulated to recapitulate the mechanical stiffness of native NB environment. NB spheroids together with human umbilical vein ECs (HUVEC) were separately incorporated into the bioprinted gelMA constructs. The NB‐EC models were cultured under static versus dynamic conditions, and we assessed the changes associated with tumorigenesis including cell viability and growth, tumor size and morphology, and NB‐EC integrations.

## 2 Results and Discussion

**Figure 1:**
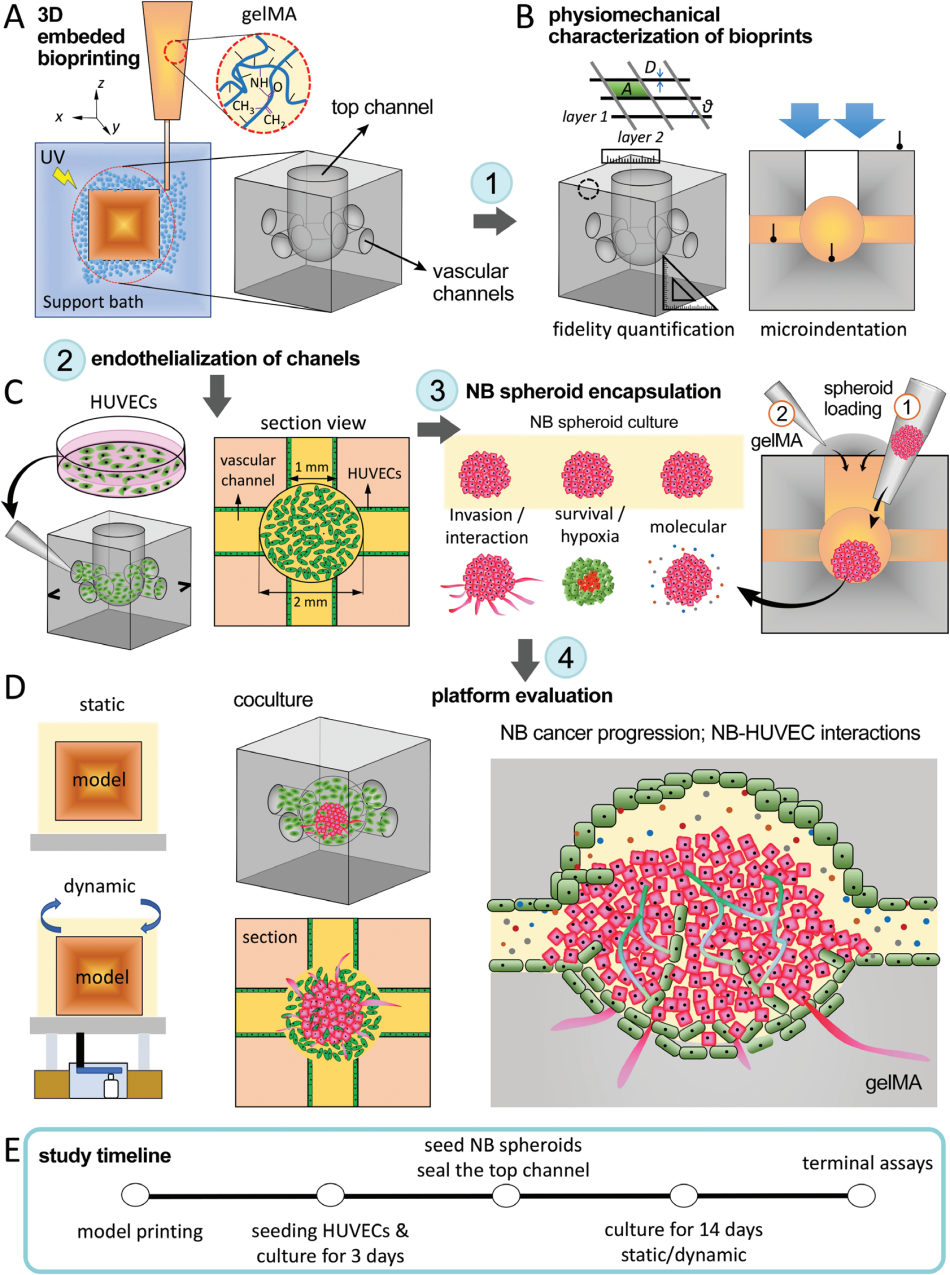
Schematic illustration of the research workflow in this study. A) 3D embedded bioprinting of gelMA tissue models, consisting of a network of interconnected channels on the lateral sides (serving as vasculature) and one channel on top (used to incorporate the NB spheroid). B) Physiomechanical characterization of bioprinted gelMA constructs. Printing fidelity was quantified through measuring several structural parameters, including the strand diameter (*D*), inter‐strand surface area (*A*), strand uniformity (*U*), and inter‐layer angle (*α*), normalized by the baseline values for each parameter in the CAD file. Mechanical properties of gelMA constructs were measured via unconfined compression as well as microindentation assays. C) Seeding HUVECs (left) and NB spheroids (right) into the 3D bioprinted constructs. HUVECs were manually seeded onto the luminal surface of side channels to form the endothelium. An NB spheroid was subsequently incorporated into the central cavity of each endothelialized gelMA construct through the top channel (step 1). This was followed by casting and sealing the top channel cavity via crosslinked gelMA (step 2). D) Evaluation of the NB‐HUVEC platforms under static versus dynamic (rocking) culture conditions. We looked at the NB tumor progression while interacting with the HUVECs. E) The timeline of the key experimental steps used in this study.

Complex tumor–TME interactions, a key characteristic of solid tumors, play significant roles in cancer progression, metastasis, and response to therapies.^[^ ^18^ ^]^ In vitro tumor models are established as valuable research enabling platform to inform our understanding of tumor‐TME interactions and treatment of various human cancer, particularly in low‐cost and high‐throughput drug screening applications.^[^ ^9^, ^18^, ^19^ ^]^ Recent studies, however, have highlighted the suboptimal capacity of the conventional 2D (monolayer) cultures to accurately predict drug sensitivity and cellular response.^[^ ^20^ ^]^ With the advancements in 3D cell culture, tissue bioprinting, and functional biomaterials, a new generation of in vitro cancer models have emerged, providing an unprecedented spatial and temporal control of the cellular and ECM structure.^[^ ^7^, ^21^ ^]^ 3D bioprinting has also enabled incorporation of perfusable vascular networks within the large tissue constructs,^[^ ^22^ ^]^ hence, allowing to simulate the highly vascularized.^[^ ^23^ ^]^ The bidirectional interactions between tumor cells and the vascular ECs within the TME contribute to tumor progression and metastasis and could, therefore, offer therapeutic potentials.^[^ ^24^ ^]^ In this study, we utilized an advanced 3D embedded bioprinting approach to incorporate NB spheroids and HUVECs within predefined vascular tissue geometries, to study the NB tumor progression and interplay with the endothelium (**Figure** **1**).^[^ ^25^ ^]^

GelMA is a photocrosslinkable natural bioink derived from gelatin^[^ ^26^ ^]^ that forms a stable hydrogel after UV exposure and provides a biocompatible niche to support cellular growth.^[^ ^27^ ^]^ Direct bioprinting of gelMA often faces inadequate printing fidelity.^[^ ^28^ ^]^ By employing a support bath that provides physical support for the extruded material, the embedded bioprinting method enables fabrication of complex 3D geometries with enhanced structural accuracy.^[^ ^28^, ^29^ ^]^ In this study, Carbopol was selected as the embedding material as it exhibits proper shear thinning property and has been shown to be biocompatible with various cell types.^[^ ^25^, ^30^ ^]^ By adjusting the concentration at 0.4% w/v, Carbopol solution was optimized in terms of its thixotropy and yield stress (Figure S1A,B, Supporting Information). As a thermosensitive material, gelMA was regulated by temperature control and printed at 23 ℃ (Figure S1C, Supporting Information). At this temperature, the bioink performs a viscoelastic behavior, with the loss tangent (ratio of loss modulus, G’’ over storage modulus, G’) value falling into the printable range.^[^ ^31^ ^]^

**Figure 2:**
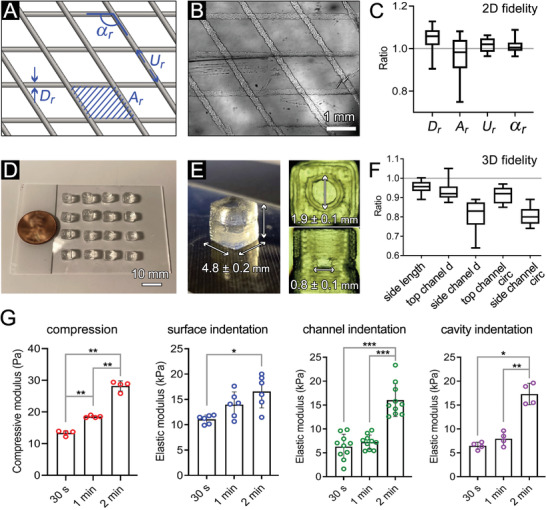
Characterization of printing fidelity and mechanical properties of bioprinted gelMA constructs. Designed (A) and bioprinted (B) two‐layer models used to assess 2D fidelity. C) Printing fidelity characterization for a two‐layer planar model (*n* = 15). D) Using the optimized bioink rheology and fidelity, high numbers of cubic vascular gelMA models were printed (16 per run). E,F) Printing fidelity characterization of the printed gelMA in 3D (*n* = 12). G) Elastic moduli of gelMA models measured from different locations at different UV exposure times. A sample size *n* = 3 was used for compressive test, *n* = 6 for indentation on top surface, *n* = 10 for indentation on the channels, and *n* = 4 for indentation on the cavity. *: *p* <0.05, **: *p* <0.01, and ***: *p* < 0.005.

Using the tuned Carbopol bath and gelMA ink, the bioprinting process was controlled to manufacture gelMA models at adequate structural fidelity (**Figure** **2** and Movie S1, Supporting Information). A constant volumetric flow rate of gelMA ink at 0.33 µL s^–1^ was regulated by the printing pressure, and the printing speed was set at 10 mm s^–1^ to ensure the printed filament with a theoretical diameter of 200 µm.^[^ ^5b^ ^]^ The two‐layer prints were first used to evaluate the printing fidelity indices, i.e., the strand diameter ratio (*D_r_*), uniformity ratio (*U_r_*), angle ratio (*α*_*r*_), and inter‐strand area ratio (*A_r_*) (ratio between actual and theoretical CAD values), which all approached 1 (ranging from 0.96 to 1.04), suggesting a high 2D fidelity (Figure 2A–C). We subsequently printed the cubic cancer models using the embedded printing approach. Since the Carbopol bath provided support for the printed, non‐crosslinked constructs, the embedded bioprinting method allowed for creating large numbers (>16) of cubic model per run (Figure 2D), demonstrating the great potential of this technique for high‐throughput tissue manufacturing.^[^ ^32^ ^]^ Printing fidelity was also assessed in the 3D cubic constructs by measuring several structural parameters (normalized by CAD values). These included the side length, top channel diameter and circularity, and side channel diameter and circularity. Results suggested relatively high consistency in the printed cube's side length (ratio of 0.95 ± 0.01), the top channel diameter (ratio of 0.93 ± 0.02), and the top channel circularity (ratio of 0.91 ± 0.01) (Figure 2E,F). However, the side channel diameter and circularity showed relatively lower accuracy, with ratios of 0.81 ± 0.03 and 0.80 ± 0.02, respectively. This reduced fidelity of the side channels, in comparison to the top, could be attributed to the effect of gravitational force in deforming the soft hydrogel channels on the lateral sides of the cube. This could be improved in future works by increasing the yield stress of embedded bath (e.g., increased Carbopol concentration), intensifying the gelMA crosslinking, and increasing printing speed.^[^ ^28^, ^33^ ^]^

Altered ECM stiffness is a key characteristic of the TME which influences the function of tumor cells at each step of cancer progression and metastasis.^[^ ^34^ ^]^ Therefore, mimicking the natural mechanical stiffness of the NB TME is essential to accurately model the cellular crosstalk.^[^ ^15^, ^35^ ^]^ By controlling the UV exposure time at constant intensity, the stiffness of the bioprinted gelMA tissues was modulated (Figure 2G, Figure S2A,B, Supporting Information). The compressive modulus of gelMA constructs significantly increased from 13.34 ± 0.76 to 28.21 ± 1.60 kPa, as the exposure period increased from 30 seconds to 2 minutes (Figure 2G–I, Figure S2A,B, Supporting Information). This stiffness range was higher than the published in vivo values for NB tissue (≈5 kPa).^[^ ^36^ ^]^ Considering the nonuniform nature of the crosslinking process, with the UV light penetrating from top through the bottom and reverse, a gradient in mechanical properties of crosslinked gelMA is expected along its height, with the middle part exhibiting the lowest stiffness. Microindentation results confirmed that the existence of a stiffness gradient in the models, with stiffer top/bottom surfaces compared to the vessel channels and the central cavity (Figure 2G). On the central cavity, the elastic modulus values were at 6.50 ± 0.76 and 7.95 ± 1.44 kPa for 30 s and 1 min UV exposure, approaching the stiffness of the natural NB tissue.^[^ ^36^ ^]^ Of note, distinct mechanical properties were quantified via unconfined compression in comparison to the microindentation method, which can be attributed to the macro versus micro‐scale of the testing conditions, respectively. Since there was no significant difference between 30 s and 1 min models in terms of stiffness, we selected the 1 min exposure, which yielded better printability, as a standard crosslinking for bioprinting the rest of gelMA models in the study.

**Figure 3:**
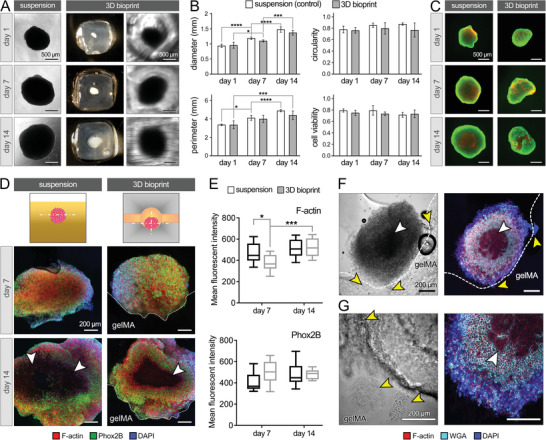
NB spheroid survival, growth, and interaction with the 3D matrix in the bioprinted gelMA construct. A) NB spheroid growth from day 1 to 14 in the suspended (control) versus 3D gelMA groups. B) Quantification of NB spheroid diameter, perimeter, and circularity, based on the optical images in A (*n* = 6 per group), and quantification of NB cell viability based on the Live/Dead assay in C (*n* = 3 per group). C) Live/dead assay conducted on the whole NB spheroids in suspension (left) versus 3D bioprints (right). Scale bars in A and C represent 500 µm. D) Immunohistochemical analysis of cultured NB spheroid at days 7 and 14, staining for F‐actin (red), Phox2B (green) and DAPI (blue). E) Quantification of F‐actin (top) and Phox2B (bottom) mean fluorescent intensity obtained from confocal images in D (*n* = 5 per group). F,G) Interaction between NB cells and gelMA at day 14 of culture, by staining for F‐actin (red), WGA (cyan) and DAPI (blue). White arrows highlight the necrotic core in the center of spheroids. Yellow arrows point to the invasion of NB cells into the gelMA matrix. Scale bars in (D), (F) and (G) represent 200 µm. *: *p* <0.05, ***: *p* < 0.005, and ****: *p* < 0.001.

We next assessed the ability of bioprinted microchanneled constructs to maintain NB spheroid viability and growth within the 3D gelMA. In situ bright field imaging of the NB neurospheres demonstrated a significant growth in the spheroid size over the 2‐week culture, both in the control group (NB spheroid suspended in NB media) and in those encapsulated in the bioprinted gelMA (1.4 to 1.6‐fold increase in diameter over 14 days, **Figure** **3**A–C). Compared to the controls, the size and shape of the NB spheroids in the gelMA, measured by diameter, perimeter, and circularity, showed reduced growth and circularity that were not significant (Figures 3B). The slight differences in shape and growth could be attributed to the interactions between NB cells and the gelMA matrix, which could alter the attachment and growth of the spheroids. Live/dead assay showed that 71–80% of cells survived in the control group and 73–75% of cells survived in printed gelMA cubes over the 2‐week culture, with no significant differences between the two groups (Figures 3B,C). These results demonstrate the ability of the bioprinted channeled constructs to maintain NB spheroid viability and growth over a 2‐week in vitro culture. The data also demonstrate the formation of size‐governed hypoxic central regions in both the suspension and bioprint‐encapsulated NB spheroids (Figure 3C), which have been shown to play key roles in the solid tumor angiogenesis, invasion, metastasis, and response to therapy.^[^ ^37^ ^]^

More in‐depth analysis of the NB spheroid structure and function within the bioprinted hydrogel models was performed via immunohistochemistry (IHC) and confocal imaging (Figure 3D–F). Filamentous actin (F‐actin), highly related to cell migration,^[^ ^38^ ^]^ and PHOX2B, a key mediator of NB differentiation and stemness maintenance,^[^ ^39^ ^]^ were used for staining. Results showed the distribution of F‐actin (red) and PHOX2B (green) throughout the spheroid sections at days 7 and 14 of culture for both control and bioprinted groups, except in the hypoxic central areas where the cell survival was low (Figure 3D,E). The F‐actin expression in the gelMA model was lower than the control group on day 7, but significantly increased to a comparable level on day 14 while the control group showed stable expression over time (Figure 3E). This suggests an initial effect of 3D gelMA encapsulation, impeding F‐actin formation and thus, the migration and expansion (invasion) of NB cells in the first 7 days but alleviated in the second week of culture. These results agree with the spheroid diameter measurements (Figure 3B) that showed a significant increase in NB size in the second week of culture. Of note, NB spheroids cultured in the bioprinted constructs did not yield an adverse effect on the PHOX2B expression in comparison to control suspended cultures (Figure 3E, bottom).

Characterizing the interactions between cancer cells and their surrounding (i.e., the TME) is one of the key steps towards understanding the process of cancer migration and invasion.^[^ ^40^ ^]^ The IHC analysis highlighted the integration of growing spheroids within the gelMA matrix, as well as formation of the hypoxic cores in the spheroids, particularly at day 14 (Figure 3D,F,G). Of note, the hypoxic core was slightly smaller in the 3D bioprints (qualitatively, white arrows in Figure 3D,F,G), which was congruent with the Live/Dead results (Figure 3C). Brightfield and confocal images also demonstrated a partial interface between the NB spheroid and gelMA, with some limited protrusions of NB cells into the 3D matrix (yellow arrows, Figure 3F,G). Together, these results confirm the capacity of the bioprinted channeled constructs to provide a compatible and supportive micro‐niche for NB tumor growth as well as a model to evaluate cellular properties contributing to invasion.

**Figure 4:**
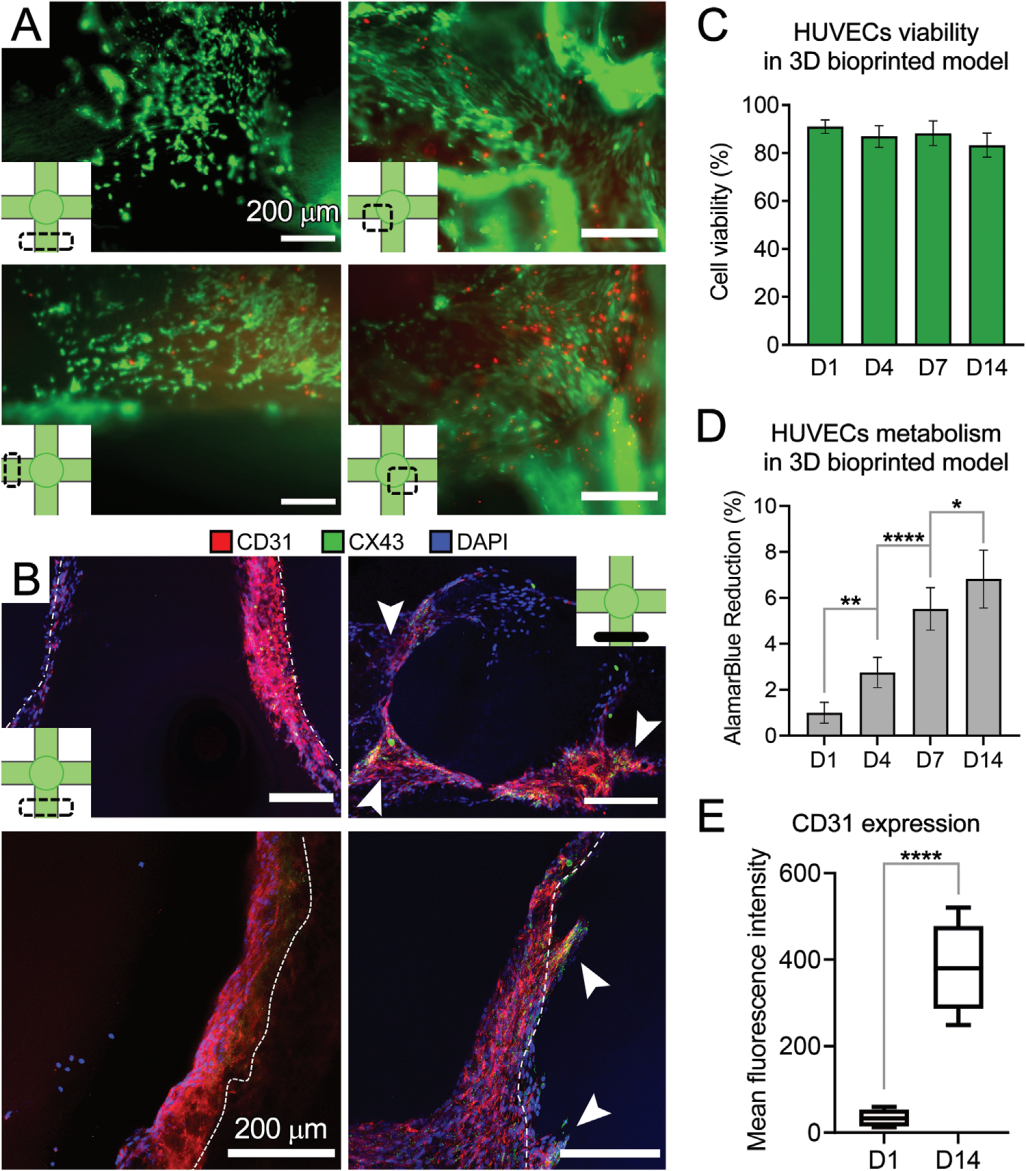
Evaluating HUVECs viability and growth within the 3D bioprinted gelMA channels. A) Live/dead results from various regions within the 3D channel structure on day 14 of culture demonstrate channel endothelialization (*n* = 3). B) CD31 (red), connexin‐43 (CX43, green) and DAPI (blue) staining of HUVECs from longitudinal (left) and tangential/perpendicular (right) views. C) Quantification of Live/Dead assay at days 1, 4, 7, and 14 of culture (*n* = 3). D) AlamarBlue assay to measure HUVECs metabolic activity, as a measure of cell viability and growth (*n* = 4). E) Quantification of the fluorescence intensity of CD31 performed on the confocal images in B (*n* = 3). Scale bars represent 200 µm. *: *p* <0.05, **: *p* < 0.01, and ****: *p* < 0.001.

Interaction between cancer cells and ECs is a key influencer on cancer invasion and metastasis.^[^ ^41^ ^]^ Cancer cells regulate tumor angiogenesis through complex direct and indirect (paracrine signaling) interactions with ECs, which promote EC proliferation, migration, and tube formation.^[^ ^42^ ^]^ To study these processes in the bioprinted model, we introduced HUVECs into the luminal space of the printed channels. Prior to testing in 3D constructs, we examined the biocompatibility of the gelMA bioink in 2D culture (Figure S3A,B, Supporting Information). The gelMA substrate supported the attachment and growth of cytoplasmic‐GFP HUVECs (> 90% viability). We subsequently loaded GFP‐labeled HUVECs into the channels of 3D bioprinted gelMA constructs. HUVECs exhibited high levels (> 80%) of viability in different zones within the 3D structure during the 14‐day culture (**Figure** **4**A–C). AlamarBlue reduction continued to increase during the culture period and showed significant increase at day 14 (p < 0.05), suggesting increased viability/proliferation of HUVECs within the bioprints (Figure 4D). IHC analysis demonstrated successful endothelialization of printed channels and central cavity in the gelMA constructs at day 14, with HUVECs expressing CD31 and CX43 (Figure 4B). Of note, HUVECs infiltrated from the channels’ luminal surface into the gelMA matrix (white arrows, Figure 4B), further confirming the bioactivity of gelMA to support EC function. The expression level of CD31 increased significantly on day 14 (p < 0.001), suggesting the sustained growth of ECs and enhanced cellular function in the gelMA bioprints (Figure 4E). The high level of endothelization and cell migration in the constructs can be attributed to the micro/macro porosities, as an inherent feature of embedded 3D bioprinting (micro‐extrusion) of hydrogel bioinks.^[^ ^5^, ^43^ ^]^ The highly porous and relatively soft scaffold architecture provides a suitable niche for ECs to attach, migrate, remodel, and secrete their own ECM.^[^ ^44^ ^]^

**Figure 5:**
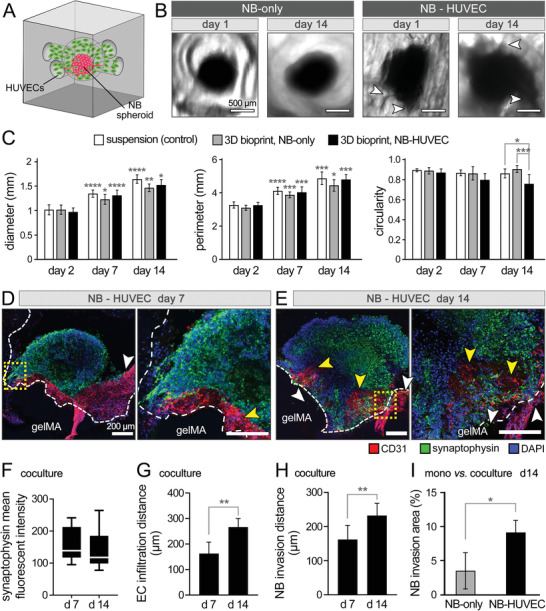
NB‐HUVEC coculture in 3D bioprinted gelMA constructs. A) Schematic illustration of the coculture. B) Bright‐field images of NB spheroids cultured for 1 and 14 days in the gelMA constructs, in the absence (NB‐only) and presence of HUVECs (NB‐HUVEC). C) Quantification of NB spheroids shape, i.e., the spheroid diameter, perimeter, and circularity of spheroids cultured in suspension (control) versus those grown in bioprinted models with or without HUVECs (*n* = 6 per group). D,E) Immunohistochemical imaging of the cocultured constructs at days 7 (D) and 14 (E). Confocal images show the staining results for EC‐specific CD31 (red), synaptophysin (green) and DAPI (blue). White arrows point to the NB invasion into the gelMA, and yellow arrows highlight EC infiltration into the spheroid. The gelMA boundary is depicted by dotted white line. F–H) Quantification of synaptophysin (F), NB cell invasion distance into the gelMA (G), and EC infiltration distance into the cancer spheroid (H), based on the confocal images (*n* = 3). I) Quantification of NB cell invasion extent into the gelMA matrix in monoculture (NB‐only) versus coculture (NB‐HUVEC) at day 14 of culture, reported as %area of gelMA tissue outside the spheroid (*n* = 3). Scale bars in (B) represent 500 µm and in (D) and (E) represent 200 µm. *: p <0.05, **: p < 0.01, ***: p < 0.005, and ****: *p* < 0.001 in comparison to the previous time point for each group.

Angiogenesis is a common and essential pre‐requisite for NB tumor progression and metastasis.^[^ ^45^ ^]^ Thus, understanding and regulating angiogenesis in a NB TME model can help develop more therapeutic strategies. Following successful endothelialization of printed channels, we investigated the coculture of NB spheroids and HUVECs in the bioprinted models. Prior to testing in 3D constructs, NB‐EC interactions were assessed on a 2D gelMA substrate, by placing a NB spheroid onto the reendothelized gelMA (Figure S3C, Supporting Information). The NB spheroids showed significant spreading onto both gelMA substrate and the 2D endothelium. HUVECs and NB cells infiltrated each other with endothelial cells migrating into the NB spheroid (Figure S3C, Supporting Information). While the 2D coculture model demonstrated adequate NB‐EC interactions, transitioning into a 3D platform which could more closely recapitulate the vascular endothelial structure and the spatial arrangement of NB and ECs in the native TME was necessary. Thus, NB‐EC coculture assays were subsequently conducted in the 3D bioprinted structures. For this purpose, HUVECs were first cultured onto the channels for 1 week, to form endothelium, followed by loading one NB spheroid into the reserved central cavity (inserted through the top channel, **Figure** **5**A). Static culture of NB‐EC loaded cancer models resulted in a sustained increase in the spheroid diameter and perimeter, which were not significantly different from those cultured in the medium (control) or in the constructs without HUVECs (NB‐only, Figure 5B). The morphology of the cocultured spheroids significantly changed after 14 days, in comparison to other two groups, with significantly reduced circularity. Considering that consistent culture media composition was used across the three groups, it can be concluded that the addition of ECs did not deter NB growth but likely altered the shape of the spheroid to an asymmetric form (Figure 5B, right). The reduced circularity in the coculture group could be explained by the crosstalk between HUVECs and NB cells, and the noticeable invasion of NB cells (Figure 3F,G) that was likely facilitated by ECs as reported elsewhere^[^ ^24^, ^46^ ^]^ and that we investigated next (Figure 5).

IHC analysis confirmed the more migratory and invasive phenotype of NB cells in the cocultured bioprints, compared to NB‐only (Figure 3D–F), with NB cells and HUVECs infiltrating each other's zones (Figure 5D–I). Cocultured NBs showed no significant difference in the NB‐specific synaptophysin expression,^[^ ^47^ ^]^ suggesting no adverse effect induced by ECs (Figure 5F). The longest travelling distance, which indexes the migratory ability of NB cells and ECs, increased significantly from day 7 to 14 in culture (Figure 5G,H). Coculture of NB cells with HUVECs also significantly increased the extent of cancer cell invasion into the gelMA matrix^[^ ^48^ ^]^ (Figure 5I). In addition, NB cells seemed to be invading the gelMA matrix through the extended EC regions (white and yellow arrows, Figure 5D,E). These results align with the characteristics of the native TME, containing cancer cells and ECs, during intravasation and metastasis,^[^ ^49^ ^]^ suggesting that the developed model can recapitulate the natural crosstalk of tumor cells and endothelium. The central hypoxic core of NB spheroids, which recapitulates the hypoxic region generated in the central area of in situ tumors, expresses various growth factors and signals to attract and recruit vascular endothelium for supplying nutrition, while cancer cells remodel the matrix, interact with ECs, and migrate.^[^ ^11^, ^50^ ^]^ This could explain the significant infiltration of HUVECs into the NB spheroids in the coculture group.

**Figure 6:**
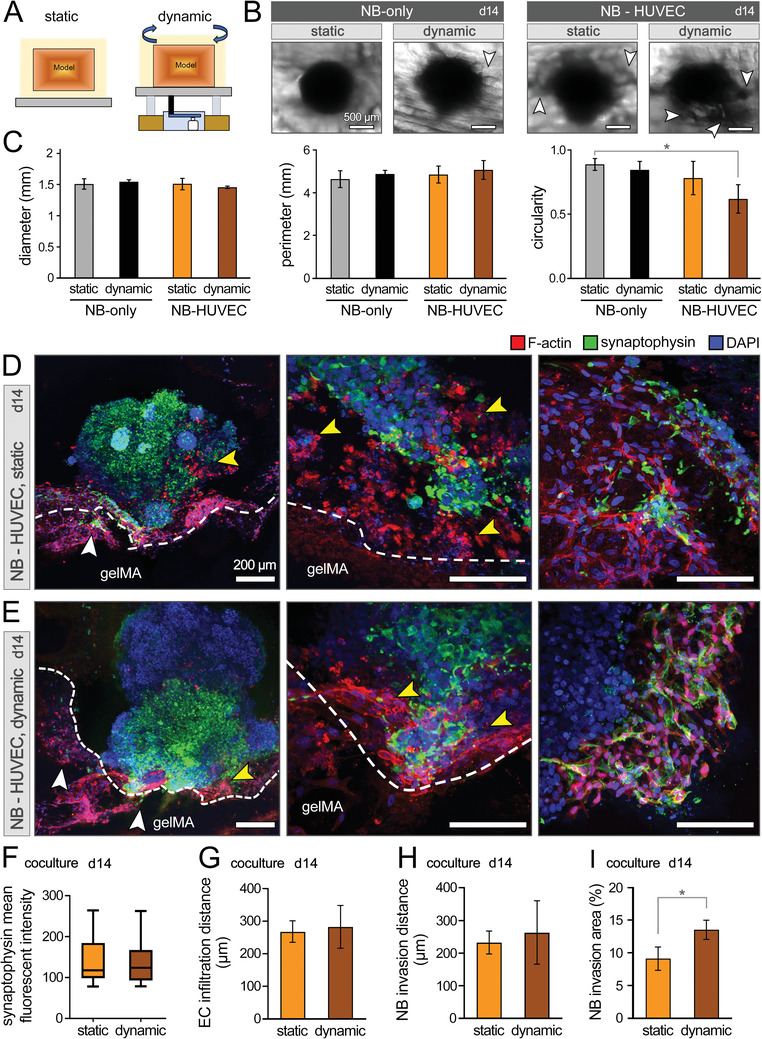
NB spheroids cocultured with HUVECs in 3D bioprinted models under static and dynamic conditions. A) Scheme of experimental set‐up for static versus dynamic (rocking) culture. B) Optical images of NB spheroid morphology in the NB‐only and NB‐HUVEC coculture groups after 14 days of static and dynamic culture. White arrows highlight the NB cell invasion to the gelMA matrix. C) Quantification of NB spheroid shape in terms of diameter, perimeter, and circularity (*n* = 6 per group). D,E) Immunohistochemical analysis of coculture models in static (D) and dynamic (E) conditions at day 14. Confocal images at different magnifications demonstrate the NB‐EC‐gelMA matrix interactions, including the NB cell invasion towards gelMA (white arrows) and EC infiltration into the NB spheroid (yellow arrows). F–I) Quantification of synaptophysin expression of NB cells (F), EC infiltration distance into the NB spheroid (G), NB cell invasion distance into the gelMA matrix (H), and NB cell invasion extent in %area of gelMA (I) at day 14 of coculture (*n* = 3 per group). Scale bars in (B) represent 500 µm and in (D) and (E) represent 200 µm. *: *p* <0.05.

Natural tumors experience dynamic microenvironments.^[^ ^51^ ^]^ The cancer model developed in this study is equipped with endothelialized microchannels, which enable the in vitro dynamic culture. Dynamic (perfused) culture allows media to enter the endothelialized channels and then flow out of the gelMA chamber housing the NB spheroid, mimicking a vessel penetrating and perfusing a solid tumor. The bioprinted NB‐HUVEC laden gelMA constructs were perfused using a rotating rocker with controlled speed to mimic dynamic vascular perfusion for 14 days (**Figure** **6**A). The diameter, perimeter, and circularity of NB spheroids showed comparable size and morphology, except for the NB‐HUVEC coculture under perfused (rocking) condition which showed significantly reduced circularity (Figure 6B,C). The diminished circularity in coculture group, which was consistent with the results presented in the static (non‐perfused) culture (Figure 5B,C), suggests that the incorporation of ECs, together with flow, may promote anisotropic migration (invasion) of NB cells (white arrows) towards the gelMA matrix.^[^ ^52^ ^]^ The inherently anisotropic ultrastructure of extrusion‐printed constructs^[^ ^53^ ^]^ could further contribute to the observed irregularity in the spheroid shape. IHC analysis of coculture groups showed remarkable infiltration of HUVECs and NB cells into the opposite territories (Figure 6D,E). While there were no significant differences in the quantified synaptophysin expression and invasion/migration distances of NB cells and ECs between static versus dynamic groups, the dynamic culture yielded increasing trends in the migratory behavior of both cell types (Figure 6F–I), where NB cell invasion extent showed significantly greater level (p <0.05) (Figure 6I). NB cells extended their protrusions from their original (cube center) location towards the gelMA matrix (white arrows, Figure 6D,E). The crosstalk between NB cells and ECs in the coculture groups was evident in the interface regions, where ECs exhibited an expansive growth deep into the growing cancer spheroid and showed evidence of new capillary formation. The invasive margins of NB cells became difficult to identify, and NB cells exhibited a trend of travelling along the established EC layer.

Together, these results suggest that the dynamic coculture group provides a more physiologically relevant TME for future studies, enabling a highly tunable dynamic flow microenvironment to assess cancer cell behavior. Both coculture of NB cancer cells with ECs, and their dynamic culture, result in significant increase in the migratory and invasive behavior of the cancer cells.^[^ ^48^ ^]^ Our model delivers a unique opportunity to study solid tumors at a single cell level to investigate molecular pathways driving cancer cell invasion (by capturing NB cells from the tumor migrating through the EC layer and into the gelMA matrix)^[^ ^54^ ^]^ as well as intravasation and extravasation (by capturing NB cells entering or exiting the endothelialized channels).^[^ ^55^ ^]^ Single cell RNA sequencing of such cells could be performed to discover novel therapeutic targets driving the aggressive metastatic phenotype.^[^ ^56^ ^]^ Furthermore, spatial proteomics and/or transcriptomics of the coculture models could shed light onto the specific cell‐cell interactions underlying NB invasion and metastasis.^[^ ^57^ ^]^ The endothelialized NB models could also serve as a high‐throughput platform to study antiangiogenic therapies that are being pursued as a method of starving tumors of their energy supply.^[^ ^45^, ^58^ ^]^

**Figure 7:**
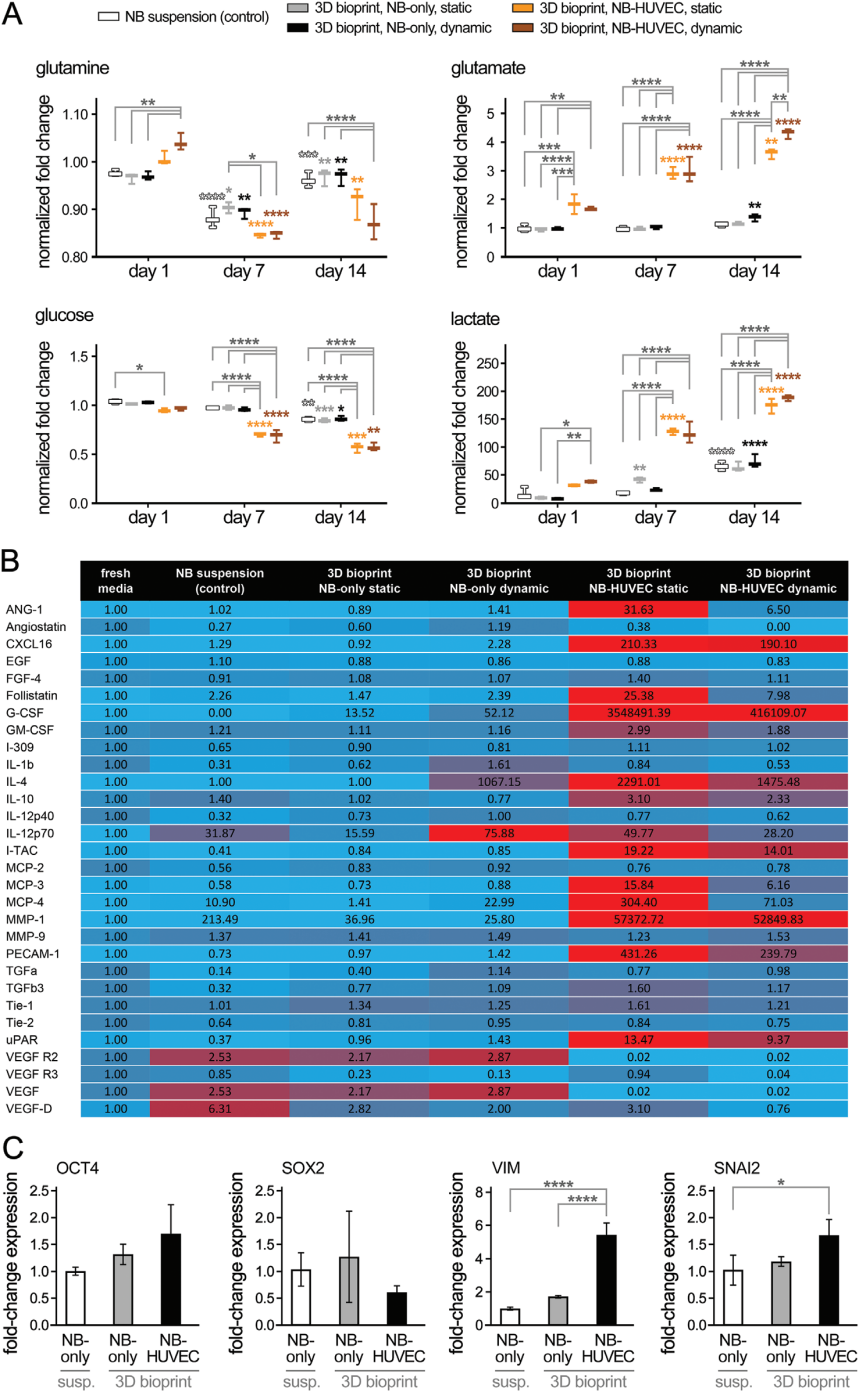
Analysis of metabolic, angiogenesis, and gene expression profiles of 3D bioprinted in vitro NB models. A) Longitudinal bioprofiling assay for nutrients (glutamine and glucose) consumption and metabolites (glutamate and lactate) accumulation, conducted on the supernatant (culture media) collected at days 1, 7, and 14 of culture (*n* = 4 per group). B) Angiogenesis analysis (heatmap) of various groups conducted at day 14 of culture. Culture supernatants were collected, incubated with conjugated antibodies, and analyzed using a microarray scanner. Samples from fresh culture media were used as control and normalization baseline (*n* = 3 per group). C) Relative gene expression (*OCT4*, *SOX2*, *VIM*, and *SNAI2*) analysis via qRT‐PCR assay conducted on the isolated NB spheroids from each condition (*n* = 3 per group). *: *p* <0.05, **: *p* < 0.01, ***: *p* < 0.005, and ****: *p* < 0.001 in comparison to the previous time point for each group.

More in‐depth functional and biological characterization of bioprinted tumor models were performed via examining metabolic activity, angiogenesis‐associated cytokines, and gene expression profiles of the incorporated cells. The longitudinal bioprofiling assay on the supernatant (culture media) showed that in comparison to the NB‐only groups (suspended control and spheroids in cubes), the NB‐HUVEC constructs, both in static and dynamic culture, exhibited significantly higher levels of nutrient metabolite consumption (glutamine and glucose) with concomitant metabolite production and accumulation (lactate and glutamate) which were normalized to the baseline media (**Figure** **7**A). The increased metabolism of the coculture groups could be attributed to the increased total number of cells (addition of ECs), as well as higher activity (growth) of the incorporated ECs. In comparison to static culture, the dynamic coculture group resulted in slightly higher levels of nutrient consumption and waste production, which became significant in the case of glutamate production at day 14 (p < 0.01). This could be attributed to the facilitated exchange of metabolites in the 3D bioprinted tumor models that were perfused (dynamic) versus the static culture. The NB cells used in these 3D bioprint models are *MYCN* amplified, an oncogene known to affect prognosis in high‐risk NB. *MYCN* is known to play an important role in regulating NB cancer cell metabolism, promoting cell growth preferentially through glutamine metabolism,^[^ ^59^ ^]^ which may explain the increased glutamate in the coculture model. Thus, the dynamic coculture group provides a more metabolically viable environment and reduces the metabolic stress and inactivity that may be experienced by models in static culture.^[^ ^60^ ^]^ Such model can be used for further investigative efforts into understanding and therapeutically targeting *MYCN* driven metabolism in NB. A laminar pulsatile flow, which can be implemented using various perfusion bioreactor systems,^[^ ^22a,b^ ^]^ would provide more physiologically relevant flow hemodynamics through the bioprinted endothelialized channels, to support NB tumor spheroids metabolic activity and growth through continuous facilitation of nutrient exchange and toxic metabolite removal.^[^ ^61^ ^]^

To further analyze the angiogenic activity of endothelialized NB constructs, the culture media supernatants were collected at day 14 from six different conditions (fresh media, NB spheroid in suspension, and NB spheroids in 3D bioprinted gelMA cultured with or without HUVECs, in static versus dynamic flow) and examined via an array of conjugated antibodies (Figure 7B). Normalized cytokine levels showed notable variations amongst these groups. Minimal differences were seen between the suspended NBs (control) and NB‐only in bioprints in static culture (columns 2 and 3, Figure 7B), with few upregulated markers in the control group, including IL‐12p70 and VEGF‐D. These changes could be attributed to the effect of NB encapsulation and NB interaction with the gelMA ECM. The comparison between static versus dynamic NB‐only in bioprints (columns 3 and 4, Figure 7B), showed significant (>50%) upregulation of ANG‐1, Angiostatin, CXCL16, Follistatin, G‐CSF, IL‐1b, IL‐4, IL‐12p70, MCP‐4, and TGFa in the dynamic culture. Among the 30 screened markers, only a few showed noticeable declines (≈30–40%) in the dynamic group, including MMP‐1 and VEGF R3. These results suggest the significant effect of the dynamic culture on promoting the angiogenic cytokines secretion in bioprinted tumor models.^[^ ^45^ ^]^ The static NB‐only versus NB‐HUVEC groups (columns 3 and 5, Figure 7B) demonstrated drastic upregulation of most angiogenic cytokines in the coculture group, including ANG‐1, CXCL16, Follistatin, G‐CSF, GM‐CSF, IL‐4, I‐TAC, MCP‐3 and 4, MMP‐1, and PECAM‐1 (showing >1000% increase). This could be related to the incorporation of ECs in the culture and their direct secretion of cytokines, as well as the effect of ECs in modulating the phenotype of the NB cells.^[^ ^62^ ^]^ VEGF R2 and VEGF were the only two markers that showed almost a 100% downregulation in coculture. Similar results were observed when comparing the cytokine levels in the dynamic NB‐only versus NB‐HUVEC coculture groups (columns 4 and 6, Figure 7B). Finally, the static coculture group showed highest levels of upregulation in angiogenic markers, against the dynamic coculture (columns 5 and 6, Figure 7B) and other study groups. Together, these results suggest that the static NB‐HUVEC culture in the 3D bioprinted gelMA models exhibited the greatest level of angiogenic activity. Higher angiogenic and proliferative profile of static coculture in comparison to dynamic coculture could be attributed to the effect of reduced/disturbed flow and shear stress in increasing vascular oxidative stress, and hence progression of proliferation and angiogenesis.^[^ ^63^ ^]^ In contrast, cells exposed to dynamic flow devote more resources in forming cell‐cell interactions and perhaps the introduction of ECs in an aggressive cancer culture can create a feedback loop driving neovascularization.

To highlight a few of the most significant cytokines profiled, MMP‐1 was observed at higher levels in all 3D culture conditions, suggesting active remodeling of ECM, i.e., the gelMA tissue.^[^ ^64^ ^]^ MMP‐1 was significantly elevated in the coculture conditions, which supports its involvement in vascular remodeling and angiogenesis.^[^ ^65^ ^]^ IL‐4 was highly upregulated in dynamic NB cultures and in coculture conditions. These results suggest that introduction of flow can trigger some cancer cells to produce the interleukin, perhaps to induce endothelial‐like cell populations to form neovascularization.^[^ ^66^ ^]^ The PECAM‐1 (CD31) molecule is involved in cell‐cell adhesion of ECs and plays an important role in neovascularization.^[^ ^67^ ^]^ As expected, higher CD31 levels were observed in the coculture conditions. G‐CSF is implicated in recruitment of cells to neovascularized vessels and its upregulation has been linked to higher levels of metastatic potential in tumors.^[^ ^68^ ^]^ Therefore, upregulation of G‐CSF in coculture bioprints suggests the importance of vascularization in the in situ 3D solid tumor environment. A search in the Open Targets database (https://platform.opentargets.org; date accessed: 19 December 2021), a platform for therapeutic target identification and validation,^[^ ^69^ ^]^ confirmed the significant role of some of the identified cytokines, such as MMP families, which had a high Open Targets association with nervous system diseases, cancers, or neoplasm.

Lastly, we performed gene expression analysis using quantitative real‐time PCR (qRT‐PCR) of isolated NB spheroids from each condition (Figure 7C). Results demonstrated that genes involved in stemness or mesenchymal properties, such as *OCT4* and *SOX2*, which were previously shown to be relevant in the NB mesenchymal phenotype,^[^ ^70^ ^]^ were stable in all groups, suggesting maintenance of the stemness properties in all conditions. Other genes critical in the epithelial‐mesenchymal transition,^[^ ^71^ ^]^ such as *VIM* and *SNAI2*, were significantly increased in the coculture conditions, supporting the more mesenchymal or aggressive phenotype of NB in the vascularized bioprinted models.

The results obtained from this study highlight the capacity of the developed model to recapitulate several key features of the native TME, including the structural complexity, mechanical properties, supporting viability and function of various cancer associated cell types, and dynamic culture conditions. Other physical properties such as hypoxia and nutrient deprivation that are commonly found in solid tumors can also be simulated to provide a facile and highly tunable platform to decipher the role of TME factors on NB aggression and to identify possible therapeutic targets. Nevertheless, there remain several important limitations that need to be addressed in these bioengineered models to achieve the optimal biomimicry and clinical relevance. Incorporation of other cancer associated cells, such as various infiltrating immune and stromal cells,^[^ ^72^ ^]^ which play key roles in cancer progression, aggression, and response to therapy would be a paramount step forward. Further, utilizing more accurate perfusion bioreactor systems to prescribe physiological flow regimens within vascular network would improve the biomimicry.^[^ ^73^ ^]^

### 2.1 Summary, Conclusions, and Future Works

Intensive research efforts dedicated to deciphering the mechanisms underlying tumor progression confirm that the interactions between cancer cells and the vascular endothelium are indispensable in remodelling the TME and cancer metastasis.^[^ ^74^ ^]^ To recapitulate such processes in vitro, advanced 3D bioprinting technologies, in combination with 3D cell culture techniques, have shown great promise in establishing platforms with high structural accuracy and reproducibility. Customized models have been successfully bioprinted with a variety of cancer types such as glioblastoma,^[^ ^11a^ ^]^ cholangiocarcinoma,^[^ ^11b^ ^]^ breast cancer,^[^ ^11d^ ^]^ and pancreatic cancer.^[^ ^10b^ ^]^ Although these models have made great progress in replicating the natural features of tumors with clinical relevance, they are often limited by the lack of perfusable vascular channels, which are essential for mimicking the dynamic TME, or physiologically relevant solid tumor size and density.^[^ ^7^, ^12^, ^21^ ^]^ Cancer cell suspension with high cell density has been used to create cancer droplets or spheroids^[^ ^75^ ^]^; however, the approach presented here is distinct from the naturally grown tumor aggregates, with limited ability to recapitulate the dynamic and vascular TME. Direct printing of 3D tumor analogues, using bioinks laden with cells, has made great strides, yet they require rigorous printing control and adjustment and can suffer significantly from suboptimal biomanufacturing efficiency and reproducibility, as well as altered cell viability and/or phenotype.^[^ ^17^, ^52^ ^]^

This study introduced an advanced bioprinting approach, based on optimized properties of the gelMA bioink and the Carbopol support bath, as well as a rigorous regulation of the bioprinting parameters. This method enabled biomanufacturing of vascular tumor models with high structural fidelity and reproducibility, providing a highly tunable microenvironment for direct incorporation of NB spheroids, endothelial, and other TME components. Results presented here demonstrate the feasibility of the bioprinted vascular model to maintain NB tumor viability, growth, aggression, and interaction with endothelium under dynamic culture conditions. NB spheroids and ECs cultured separately in the gelMA constructs demonstrated high viability and maintained their function. When cocultured, NB‐HUVECs exhibited enhanced cell‐cell interaction with significant NB cell migration/aggression towards the gelMA tissue and EC infiltration towards the hypoxic regions of the tumor. Metabolism, cytokine, and gene expression analyses suggest that the NB‐EC coculture model in the dynamic conditions most effectively reflects the interactions between tumor cells and vasculature.

Future studies can utilize this bioengineered platform to fully characterize the biologic properties of NB (and other) tumors in response to spatiotemporal variations in the TME parameters such as immune and stromal cells,^[^ ^76^ ^]^ hypoxia,^[^ ^77^ ^]^ angiogenesis,^[^ ^45^ ^]^ and ECM stiffness^[^ ^15^, ^35^ ^]^ and composition.^[^ ^78^ ^]^ Such models could be leveraged to study cancer onset, progression, and metastases in a reproducible manner, opening the door for high‐throughput drug screening platforms and testing of novel treatment options. In particular, integrating immune and stromal cell components of NB TME in the future generation of this model would provide a unique opportunity to study the multifaceted molecular, cellular, and immune system interactions underlying NB response to immunotherapies that are commonly used to treat high‐risk patients. The perfused in vitro models of NB would enable simulating a variety of chemotherapy, immunotherapy, and even cellular therapy combinations in order to characterize and target mechanisms of resistance to such therapies. This is especially important given that immunotherapy and chemotherapy combinations have significantly improved clinical outcomes for high‐risk NB patients yet biomarkers of response remain to be determined.^[^ ^79^ ^]^ Implementation of physiologically relevant vascular flow regimens within bioprinted endothelialized channels allows for in depth mechanistic studies while controlling tumor blood flow determinants.^[^ ^80^ ^]^ Furthermore, there may be an opportunity to utilize the platform for ex vivo studies^[^ ^81^ ^]^ by direct incorporation of extracted tumor tissues, obtained from patients, into the bioprinted models.

## 3 Experimental Section

### Materials

Gelatin from porcine skin (Type A, SLCC7838), methacrylic anhydride (MA), Irgacure (2‐Hydroxy‐4′‐(2‐hydroxye‐thoxy)‐2‐methylpropiophenone), and PBS were obtained from Sigma‐Aldrich (Wisconsin, U.S.A.). AlamarBlue and qPCR kits were purchased from Bio‐Rad (Hercules, U.S.A). Carbopol ETD 2020 polymer was purchased from Lubrizol (Wickliffe, U.S.A.). Calcein AM and propidium iodide (PI) were obtained from Biotium (Fremont, U.S.A.). HUVEC culture medium (VascuLife VEGF) was purchased from Lifeline Cell Technology (Oceanside, U.S.A.). Conjugated F‐actin and wheat germ agglutinin (WGA) were purchased from ThermoFisher, USA; CD31, Connexin 43, Phox2B and Synaptophysin antibodies were purchased from Abcam, USA.

### GelMA Synthesis, Preparation, and Evaluation

GelMA was produced from porcine gelatin (Sigma) following the developed protocol.^[^ ^25a^ ^]^ Briefly, gelatin powder was fully dissolved at 10% w/v into PBS at 50 °C. MA (Sigma) was added dropwise for gelatin modification at 50 °C for 3 h. Following a dilution with additional warm PBS to stop the reaction for 10 min, the mixture was dialyzed against deionized water for 1 week at 40 °C, with water change 2–3 times a day. The solution was then lyophilized and stored away from light at −20 °C until use. The 10% w/v gelMA solution was created by reconstituting lyophilized gelMA powder into sterile PBS with 0.5% w/v Irgacure (Sigma). GelMA solutions were stored away from ambient light at 4 °C for no longer than 2 weeks. Before use, the rheological properties of prepared gelMA solution were evaluated based on a shearing (shear rates from 0 to 1000 s^–1^) and a temperature (4–30 ℃) sweep using a cone‐and‐plate rheometer as previously described^[^ ^28b^ ^]^ (AR‐G2 rheometer, TA Instruments, *n* = 3).

### Embedded 3D Bioprinting of gelMA Constructs; Printing Fidelity Assessment

Carbopol (Lubrizol) was selected as the supporting bath material for embedded bioprinting.^[^ ^28b^ ^]^ Following the method developed previously,^[^ ^25a^ ^]^ a 0.4% w/v sterile Carbopol suspension was prepared and stored at room temperature for no more than 2 weeks. The yield stress and thixotropic behavior of Carbopol suspension was assessed using the rheometer with the measurements repeated three times^[^ ^28b^ ^]^ (*n* = 3). All embedded bioprinting works were conducted using a BioX 3D bioprinter (CELLINK, US). A needle gauge of 27 was used to ensure the adequate printing resolution. The printing pressure was controlled at 25 kPa according to the rheological properties of gelMA solution to maintain the relatively constant flow rate of 0.3 µL s^–1^.^[^ ^28b^ ^]^ GelMA ink was directly deposited into the Carbopol bath to create constructs following the designed models, which included a two‐layer lattice structure (for fidelity assessments) and the 3D tumor model. Once printed, the constructs were exposed to the UV light at 10 mW cm^−2^ with tunable exposure time for crosslinking.

Printed strand diameter ratio (*D_r_*), uniformity ratio (*U_r_*), angle ratio (*α*_*r*_), and inter‐strand area ratio (*A_r_*) were employed as indices for printing fidelity assessment in the two‐layer (2D) structures. These indices were defined as the ratio between the experimental (printed) over the theorical (CAD) value of each parameter, where the ratio of ≈1 indicates an ideal structural fidelity (*n* = 15). Similarly, the printing (3D) fidelity of NB cancer model was assessed based on the key structural factors of the model, including the cubic structure length, diameter, and circularity of both top and side channels. The ratio between the printed versus design values were calculated to evaluate the 3D fidelity (*n* = 12).

### Mechanical Tuning and Characterization of 3D Bioprinted gelMA Models

After bioprinting, the gelMA constructs, preserved in the Carbopol bath, were directly crosslinked by UV exposure from both top and bottom sides by flipping the samples (identical top and bottom crosslinking), using a constant light intensity (10 mW/cm^2^) and varied crosslinking durations, including 30 s, 1 min, and 2 min. After PBS wash, the stiffness (*S*) and elastic modulus (*E*) of gelMA constructs were measured using both unconfined compression and microindentation tests, using the Mach‐1 mechanical testing system (Biomomentum Inc., Quebec, Canada) as previously described.^[^ ^22^, ^28^, ^82^ ^]^ Unconfined compression was applied on samples with 50% strain in total at 20 µm s^–1^. The compressive modulus was derived from the slope of the linear tread line (initial 0–20%) of the stress–strain curve (*n* = 3). To assess the local mechanical properties of gelMA model, microindentation was performed on the surface of the model (*n* = 6), the inner surface of the microchannels (*n* = 10), and on the central cavity (*n* = 4). A 500 µm spherical indenter was utilized, with the indenting depth of 100 µm at 2 µm s^–1^. The force‐displacement unloading curves were plotted and used to calculate the stiffness of the sample (*S*) from the linear tread line slope (initial 5–20%), and the reduced elastic modulus (*E_r_*) was derived following the formula.^[^ ^83^ ^]^

(1) $$E_{r}=\frac{\sqrt{\pi }}{2\beta }\frac{S}{\sqrt{A\left(h_{c}\right)}}$$

where *β* is a constant and equals 1, *A*(*h_c_*) is projected contact area at the contact depth of *h_c_*. It can be obtained from the following equation:

(2) $$A\left(h_{c}\right)=2\pi Rh_{c}-\pi {h_{c}}^{2}$$

where

(3) $$h_{c}=h_{\max}-\varepsilon \frac{P_{\max}}{S}$$

where *h*_max_ and *P*_max_ are the peak unloading displacement and unloading force, respectively, and *ε* is a constant with a value of 0.75 for a spherical indenter.^[^ ^84^ ^]^

The elastic modulus, *E*, can be calculated using the following equation.^[^ ^83^ ^]^

(4) $$\frac{1}{E_{r}}=\frac{\left(1-v^{2}\right)}{E}+\frac{1-v_{i}^{2}}{E_{i}}$$

where *v* is the Poisson's ratio of tested material with a value of 0.5, and *v_i_* is 0.5 for the indenter tip material. *E_i_* represents the elastic modulus of the probe, with a value of 2 GPa.

### Preparation of NB Cell Lines; 3D Culture of NB Spheroids

Human‐derived NB cell line, IMR5, was obtained from the Children's Oncology Group Childhood Cancer Repository. NB cells were cultured in RPMI‐1640 (Sigma) with 10% fetal bovine serum (Gemini), 1% penicillin:streptomycin (Gemini), and incubated at 37 ℃ with 5% CO_2_.^[^ ^85^ ^]^ All cells used were maintained at low passage, not exceeding 15 passages. Cell lines underwent STR‐based genotyping (Texas Tech Cancer Cell Repository) and identities were verified using the COG cell line genotype database (www.cccells.org). Cells were tested for Mycoplasma routinely with Mycoalert Mycoplasma Detection Kit (Lonza). NB cells were plated at 30000 cells per mL in neurobasal media [50/50 F12/DMEM (Thermo Fisher Scientific), 1x B27 (Thermo Fisher Scientific), 1x N2 (Thermo Fisher Scientific), 0.1 mm ΒME (Sigma), 2 µg mL^−1^ heparin (Stemcell Technologies), 1% penicillin:streptomycin (Gemini), 20 ng mL^−1^ EGF (Corning), 40 ng mL^−1^ FGF (Corning)] and cultured in 6‐well ultra‐low attachment plates (Corning) for 10 days. Single spheroids were selected out and each placed in a well of 96‐well ultra‐low attachment plates until reached 1000 µm in diameter.

### Loading NB Spheroids into 3D Bioprinted gelMA Constructs; Tumorigenesis Assays

Spheroids at a diameter of 1000 µm were manually loaded (via a pipet) through the top channel into the cavity of the bioprinted gelMA constructs. Prior to seeding, the gelMA model was thoroughly coated with 0.5% gelatin for 24 h to enhance cell affinity. After spheroid loading, a 10 µL 10% w/v gelMA solution was cast into the top channel and crosslinked immediately at 10 mW cm^−2^ for 30 s to ensure sealing of that channel. The spheroid‐loaded gelMA constructs were moved to 24‐well plates with neurobasal media for culture. NB spheroids in suspension were used as control and cultured in 24‐well plates. Multiple measurements were conducted for NB spheroid characterization. On days 3, 7 and 14, bright‐field images were taken via an epifluorescence microscope (Leica Microsystems) from NBs in both the gelMA tissue and those in suspension (*n* = 6). These images were analyzed to quantify and the size and shape of the NB spheroids in terms of diameter, perimeter, and circularity, which helped to assess the growth of the spheroids in different culture groups.

Cell viability was evaluated using Live/Dead assay as described before.^[^ ^86^ ^]^ Briefly, fluorescent dyes of 1 µg mL^−1^ calcein‐AM and 20 µg mL^−1^ propidium iodide (PI) (Biotium) were added to the culture media to selectively stain live (green) and dead (red) cells, respectively. After 20 min incubation, substrates or scaffolds were rinsed with fresh culture media and imaged by fluorescence microscopy (Leica Microsystems). Three randomly selected image fields were evaluated using ImageJ for each construct at each time point (*n* = 3).

IHC analysis was conducted on days 7 and 14 of culture. GelMA constructs and control spheroids were rinsed with PBS and subsequently fixed with 10% buffered formalin. The fixed samples were transferred into 10% gelMA in a 48‐well plate for embedding before sectioning. A Leica vibratome system was used to section the cast samples into 300 µm slices, which were then blocked with 2% bovine serum albumin (BSA), 5% donkey serum, and 0.2% Triton in PBS for 2 h. Rabbit anti‐Phox2B (1:200) in PBS was added for 4 h at room temperature, and samples were washed three times in PBS (room temperature for 1 h; 4 ℃ overnight; and room temperature for 2–3 h). Samples were then incubated with Alexa Fluor 488 rabbit anti‐donkey antibody (1:200), conjugated 594 F‐actin (1:200), 647 WGA (1:500), and DAPI (1:1000) in PBS for 4 h at room temperature and washed a second time following the same procedure as described. Slices were mounted and images were captured using a confocal fluorescence microscope (FV1000, Olympus). For each group, three samples were prepared, and three random images from each sample were taken for fluorescence intensity measurement using ImageJ (*n* = 3).

### Endothelialization of Bioprinted Channels and Their Characterization

HUVECs (ATCC) were cultured in tissue culture flasks in a tissue culture incubator (37°C with a 5% CO_2_) using VEGF Endothelial Medium Complete Kit (VascuLife). A cell passage number of 17 was used for all 3D bioprinting assays. After harvesting at 90% confluency, HUVECs were manually pipetted into the gelatin pre‐coated channels in the gelMA construct at a density of 10^7^ cells mL^−1^. For this purpose, cells were distributed equally into the four lateral channels of the cube. After 2 h incubation (to ensure cells attachment), the EC‐seeded gelMA models were transferred into 24‐well plates for the continuation of culture.

Live/Dead assay was conducted to assess cell viability on days 1, 7, and 14 as described above. Following the same process described, bright‐field images were taken randomly from samples and were evaluated using ImageJ at each time point (*n* = 3) to quantify spheroid growth. AlamarBlue assay was also employed following the previous works.^[^ ^87^ ^]^ Briefly, the AlamarBlue reagent (Bio‐Rad) was added to fresh HUVEC culture medium in a 1:9 volumetric ratio and added to the culture plates containing the samples. After 5 h incubation, a 100 µL of the media was collected and placed in a 96‐well plate for readout. The absorbance was read using a microplate reader (BioTek Instruments, USA) at the wavelengths of 550 and 600 nm. Percentage of AlamarBlue reduction were calculated on days 1, 4, 7, and 14 and normalized by the day 1 as baseline (*n* = 4).

IHC was performed following the same procedures as above, while EC‐specific primary rabbit anti‐CD31 (1:200) and mouse anti‐Connexin 43 (1:200) antibodies, and secondary Invitrogen 647 rabbit anti‐donkey antibody (1:200) and 568 mouse anti‐donkey antibody (1:200) were used alternatively. CD31 fluorescent intensity was quantified using three randomly taken confocal images from each group (*n* = 3) at day 1 and 14.

### NB Spheroid‐HUVEC Coculture and Tumorigenesis Assays

HUVECs were first seeded onto the channel surfaces of bioprinted gelMA at a density of 10^7^ cells mL^−1^, as described above, followed by a 3‐day culture in the EC media. At day 4, a NB spheroid was manually loaded through the top channel followed by sealing the channel with gelMA. The prepared multicellular models were cultured in a mixed culture media containing 50:50 HUVEC and neurobasal media for 14 days. Bioprinted gelMA constructs containing only the NB spheroids (NB‐only) were simultaneously prepared as control, while the 50:50 media was still used for their culture to maintain consistency across study groups. The shape and size of NB spheroids in terms of diameter, perimeter, and circularity were assessed following the steps described above based on bright‐field imaging method (*n* = 6).

IHC was employed to assess the function of the two cell types and their interactions. Rabbit anti‐Synaptophysin (1:200) and mouse anti‐CD31 (1:200) were used as primary antibodies to selectively bond to NB cells and HUVECs, followed by the addition of secondary Invitrogen 647 rabbit anti‐donkey antibody (1:200) and 568 mouse anti‐donkey antibody (1:200). The fluorescent intensity of NB spheroids, the migration of NB cells into EC layer and gelMA model, and the infiltration of ECs towards the NB spheroid (endothelial sprouting, based on the longest distance of ECs traveled from the gelMA surface towards the center of NB spheroid) were evaluated based on the obtained confocal images randomly taken from three different samples (*n* = 3).

### Static versus Dynamic Culture

GelMA constructs containing NB spheroid and HUVECs were cultured under either a static or a dynamic condition. For the dynamic culture, tissue culture plates containing the gelMA models were placed and cultured on a rocker inside the tissue culture incubator with a constant rotating speed of 30 rpm. Models that included only NB spheroid (NB‐only) were prepared as control. At day 14, samples were fixed, sectioned, stained, and assessed in terms of NB cell fluorescent intensity, NB cell migration (invasion), and EC infiltration (*n* = 3).

### Gene Expression Analysis

qRT‐PCR assay was used to analyze gene expression profile of NB spheroids. Three groups, including the NB spheroids suspended in culture media (control), bioprinted gelMA containing NB spheroids (NB‐only), and gelMA constructs containing NB spheroid‐HUVEC coculture were cultured in static condition. NB spheroids, either in media or in the gelMA models, were carefully removed via a pipette for RNA extraction using the Aurum Total RNA Mini Kit (Bio‐Rad Laboratories). RNA was reverse transcribed into cDNA using the SuperScript VILO cDNA Synthesis Kit (Thermo Fisher Scientific). qPCR was performed on an Applied Biosystems 7500 real time PCR system (Thermo Fisher Scientific) using the iTaq SYBR Green PCR master mix (Bio‐Rad Laboratories). The relative gene expression was calculated using the 2^−ΔΔCt^ method with normalization to the Ct of the housekeeping gene glyceraldehyde 3‐phosphate dehydrogenase (*GAPDH*). Multiple NB‐specific primer sequences (Integrated DNA Technologies) were used (Table S1, Supporting Information). Gene expression was assessed at day 7, with each group having three replicates (*n* = 3 per experimental group).

### Bioprofiling Analysis

At serial time points during culture (days 1, 7, and 14), 400 µL of culture media supernatants were collected from suspended NB spheroids, NB‐only in bioprinted gelMA in static versus dynamic, and NB‐HUVEC coculture samples in static versus dynamic culture (*n* = 4 per experimental group). Supernatant samples were analyzed for various metabolite contents using a NovaFlex Bioprofile 2 (NovaBiomedical), hence, quantifying the nutrients (glutamine and glucose) consumption and metabolites (glutamate and lactate) accumulation rates in different culture groups. The data were normalized to the readout for fresh media samples (baseline) to demonstrate production/consumption of metabolites as a function of culture time. Cumulative changes in metabolite production/concentration were determined and compared across each individual sample.

### Angiogenesis Microarray Analysis

The angiogenesis assay kit (Human Angiogenesis Array Q3; RayBiotech, Peachtree Corners, USA) was used following the protocol provided by the manufacturer with the following modifications. Samples were 100 µL of culture supernatant stored at −80 ℃ and thawed immediately before use. Primary supernatant incubation was carried overnight at 4 ℃ as per the optional protocol step to ensure robust binding of the target cytokines and soluble factors. Conjugated antibody incubation was also carried out overnight at 4 ℃ to maximize signal strength and minimize signal to noise ratio. Completed slide for the assay was fully dried at the last step of the process and stored at 4 ℃, protected from light until analysis. Readouts were performed on an Innoscan 1100AL microarray scanner (Innopsys, Chicago, IL, USA). We analyzed five unique cultures: NB suspension (control), NB‐only in bioprinted gelMA in static or dynamic culture, and NB‐HUVEC coculture in bioprinted gelMA in static or dynamic culture. We also included a sample of the fresh media in the analysis that served as control and normalization baseline.

### Statistical Analysis

Experimental data were processed and expressed using mean values ± standard deviation (SD). Statistical significance was determined by ordinary t test, one‐way or two‐way analysis of variance, and multiple comparisons were performed and corrected by Tukey test using GraphPad Prism with an acceptable significance level of *p* < 0.05. In the entire study, *: *p* <0.05, **: *p* < 0.01, ***: *p* < 0.005, and ****: *p* < 0.001 in comparison to the previous time point for each group. Sample size (*n*) has been presented in each experimental section in Section 3.

## Conflict of Interest

The authors declare no conflict of interest.

## Supporting information

Supporting Information

Supplemental Movie 1

## Data Availability

The data that support the findings of this study are available from the corresponding author upon reasonable request.
