# Supplementary material for: A 3D Bioprinted in vitro Model of Neuroblastoma Recapitulates Dynamic Tumor‐Endothelial Cell Interactions Contributing to Solid Tumor Aggressive Behavior
Source: Adv Sci (Weinh). 2022 May 29;9(23):2200244. doi: 10.1002/advs.202200244 (PMC9376856; doi:10.1002/advs.202200244)
Supplement: Supplementary file 1 — Supporting Information [file ADVS-9-2200244-s002.pdf]

ADVANCED SCIENCE
Open Access

Supporting Information

for *Adv. Sci.*, DOI 10.1002/advs.202200244

A 3D Bioprinted in vitro Model of Neuroblastoma Recapitulates Dynamic Tumor-Endothelial Cell Interactions Contributing to Solid Tumor Aggressive Behavior

Liqun Ning, Jenny Shim, Martin L. Tomov, Rui Liu, Riya Mehta, Andrew Mingee, Boeun Hwang, Linqi Jin, Athanasios Mantalaris, Chunhui Xu, Morteza Mahmoudi, Kelly C. Goldsmith and Vahid Serpooshan**

A 3D Bioprinted *in vitro* Model of Neuroblastoma Recapitulates Dynamic Tumor-Endothelial Cell Interactions Contributing to Solid Tumor Aggressive Behavior

Liqun Ning, Jenny Shim, Martin L. Tomov, Rui Liu, Riya Mehta, Andrew Mingee, Boeun Hwang, Linqi Jin, Athanasios Mantalaris, Chunhui Xu, Morteza Mahmoudi, Kelly C. Goldsmith*, and Vahid Serpooshan*

Dr. L. Ning, Dr. M. Tomov, A. Mingee, B. Hwang, L. Jin, A. Mantalaris, Dr. C. Xu, and Dr. V. Serpooshan
Wallace H. Coulter Department of Biomedical Engineering, Emory University School of Medicine and Georgia Institute of Technology, Atlanta, GA, United States

Dr. J. Shim, Dr. R. Liu, Dr. C. Xu, Dr. K. Goldsmith, and Dr. V. Serpooshan
Department of Pediatrics, Emory University School of Medicine, Atlanta, GA, United States

Drs. J. Shim and K. Goldsmith
Aflac Cancer and Blood Disorders Center, Children's Healthcare of Atlanta, Atlanta, GA, United States

Drs. J. Shim, Dr., K. Goldsmith and V. Serpooshan
Children’s Healthcare of Atlanta, Atlanta, GA, United States

R. Mehta
Department of Biology, Emory University, Atlanta, GA, United States

Dr. M. Mahmoudi
Department of Radiology and Precision Health Program, Michigan State University, East Lansing, MI, United States

Drs. L. Ning and J. Shim contribute equally to this study

* Corresponding authors: vahid.serpooshan@bme.gatech.edu; kgoldsm@emory.edu

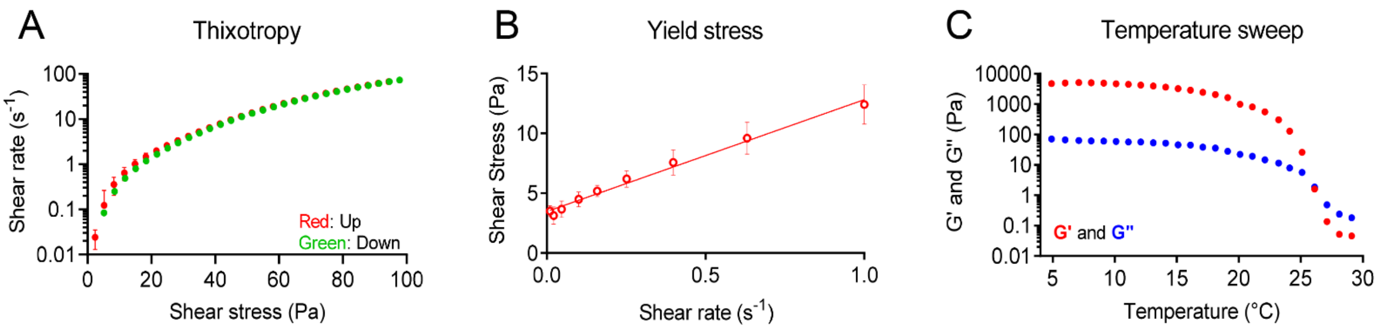

Figure S1. **Rheological analysis of Carbopol support bath and gelMA bioink**. **A**: Thixotropy results of 0.4% w/v Carbopol bath. The hysteresis loop was plotted based on an increase-decrease control on shear stress between 0 to 100 Pa. The loop demonstrates limited thixotropic time with almost overlapping up and down curves, indicating the ability of Carbopol bath to rapidly recover under shearing. **B**: Yield stress of 0.4% Carbopol bath, achieved by applying a range of small shear rates from 0.1 to 1 s^{-1}, and then interpolating the obtained shear stress at zero share rate. The yield stress is 3.48 Pa. **C**: Temperature sweep to identify the gelling point of 10% w/v gelMA. Lower temperatures increased storage (G’) value, while moving to high temperatures produced a more liquid-like solution with increased loss (G’’) value. The gelling/liquefying point was identified as ~26°C. These results ensured the printability of the bioink printed at room temperature in this study.

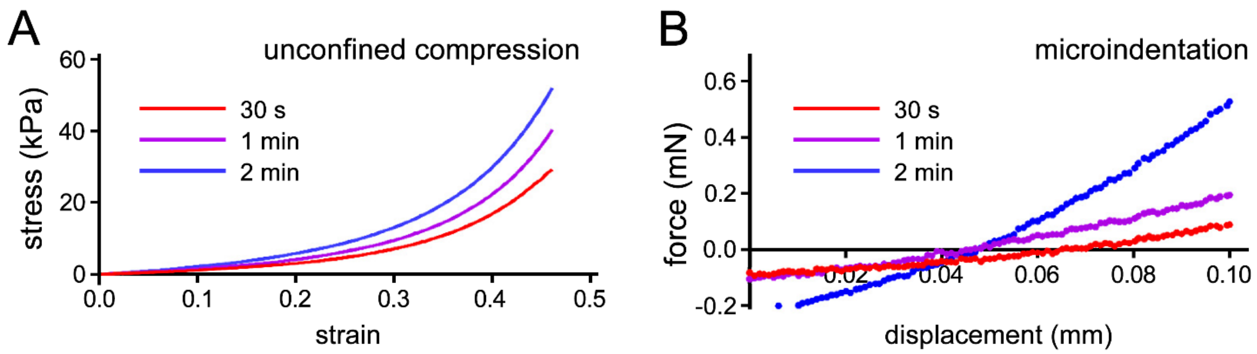

Figure S2. **Characterization of printing mechanical properties of bioprinted gelMA constructs**. **A-B**: Stress-strain curves obtained from unconfined compression test (**A**) and unloading force-displacement curves obtained from microindentation test (**B**) at different UV exposure times (30 s, 1 min, and 2 min).

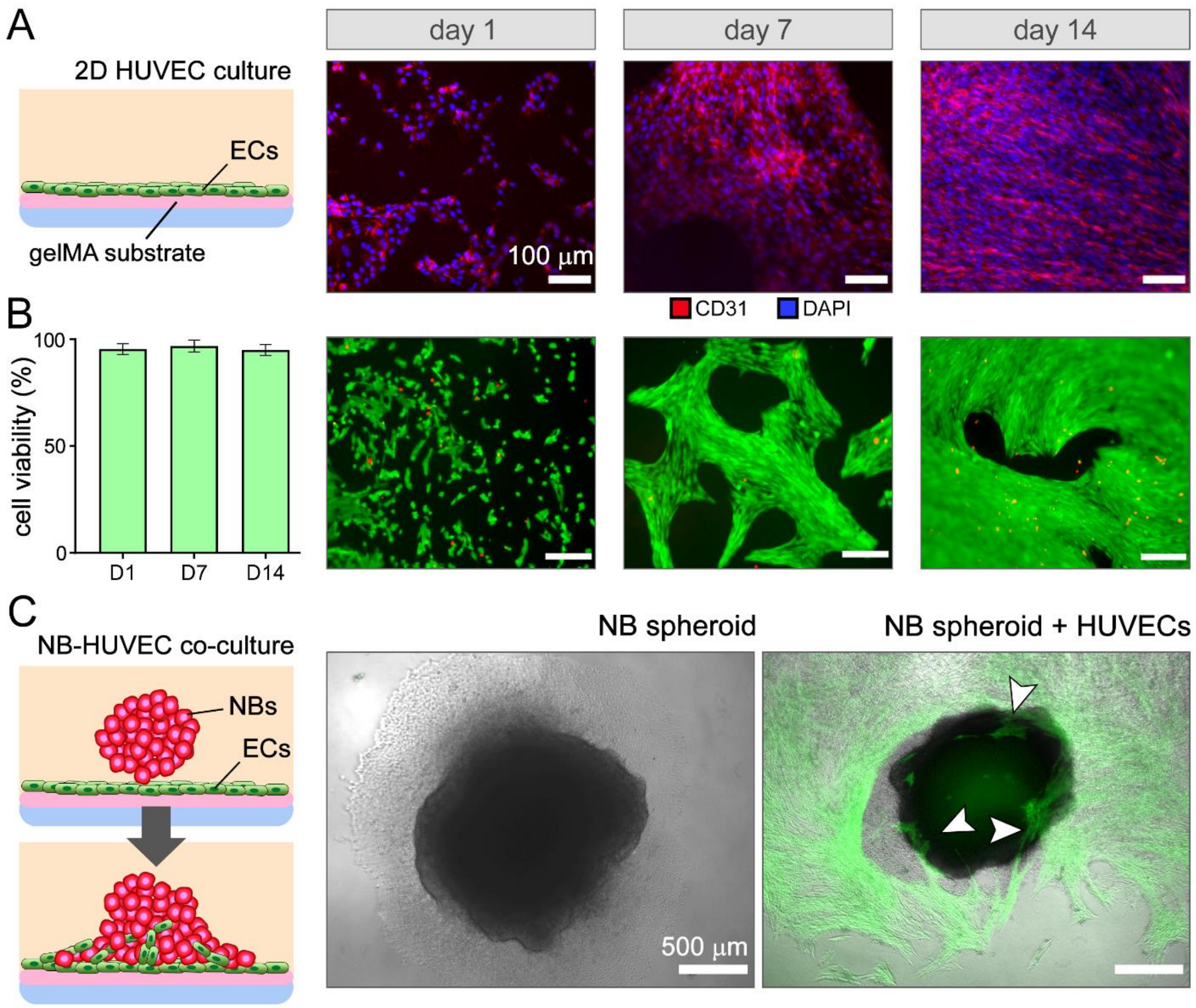

Figure S3. **Interactions between human umbilical vein endothelial cells (HUVECs), gelMA substrate, and neuroblastoma (NB) cells**. **A-B**: 2D culture of cytoplasmic-GFP HUVECs on the cast gelMA substrate. **A**: CD31 and DAPI staining of GFP HUVECs demonstrates high affinity of the cells to the gelMA bioink and their rapid growth. **B**: Live/dead assay on HUVECs at days 1, 7, and 14 of culture (*n* = 3). Scale bars in **A** and **B** represent 100 µm. **C**: Coculture of NB spheroid, loaded onto the endothelial layer grown on gelMA substrate (as in **A-B**). NB and HUVECs showed spreading and infiltration into each other. White arrows highlight penetration of HUVECs into the NB spheroid. Scale bars in **C** represent 500 µm.

Table S1: List of primers used in the qRT-PCR analysis of NB spheroids extracted from 3D bioprinted gelMA constructs.

Primers (human)	Forward (5'-3')	Reverse (5'-3')	Blast confirmed
GAPDH	GAGTCAACGGATTTGGTCGT	GACAAGCTTCCCGTTCTCAG	YES
OCT4	GGAGGAAGCTGACAACAATGAAA	GGCCTGCACGAGGGTTT	YES
SOX2	TGCGAGCGCTGCACAT	TCATGAGCGTCTTGGTTTTCC	YES
VIM	GACAATGCGTCTCTGGCACGTCTT	TCCTCCGCCTCCTGCAGGTTCTT	YES
SNAI2	CAGACCCTGGTTGCTTCAA	TGACCTGTCTGCAAATGCTC	YES

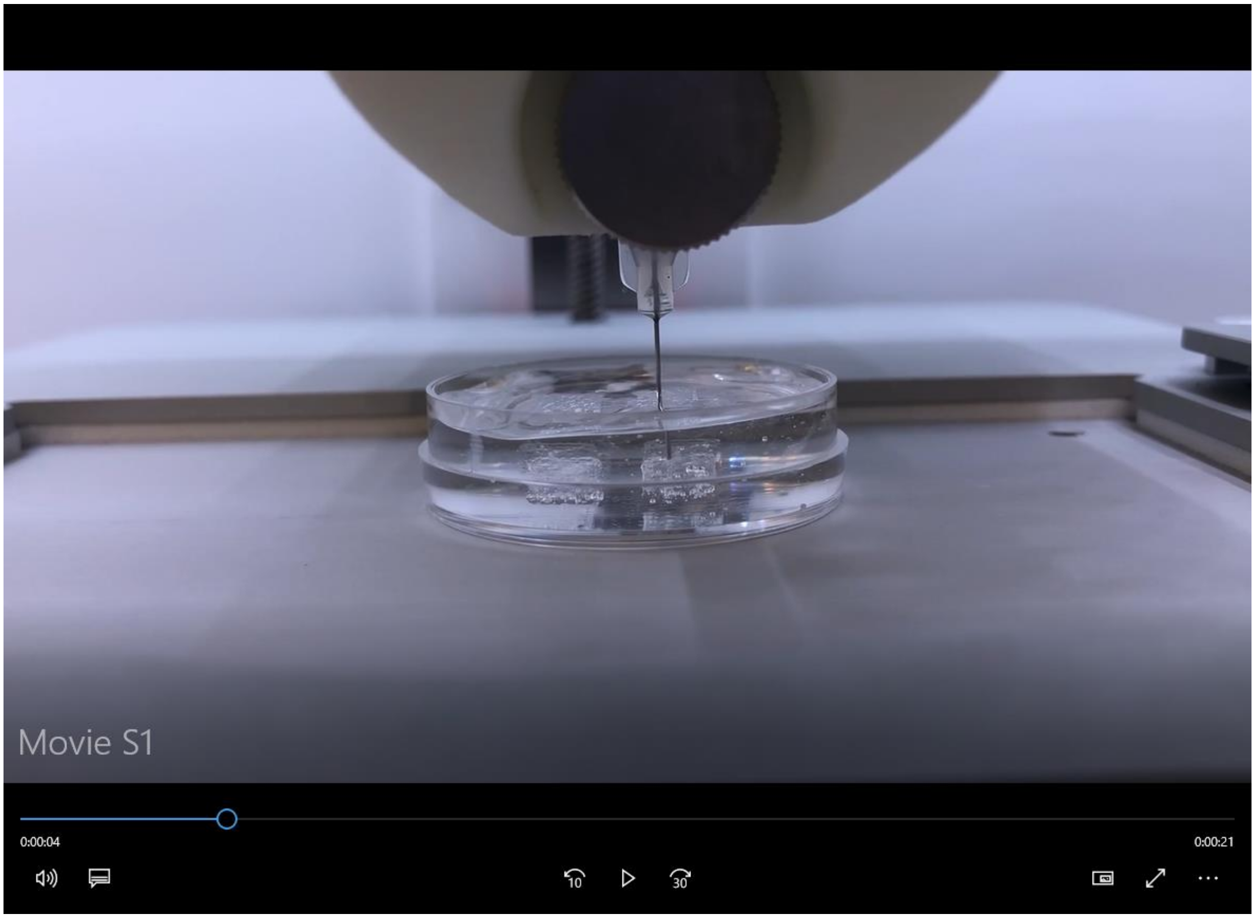

Movie S1: Representative bioprinting process used to simultaneously manufacture multiple vascular gelMA constructs.
